# Recent Advances in Porphyrin-Based Covalent Organic Frameworks for Synergistic Photodynamic and Photothermal Therapy

**DOI:** 10.3390/pharmaceutics16121625

**Published:** 2024-12-22

**Authors:** Cheng Qi, Jiayi Chen, Yijie Qu, Xuanxuan Luo, Weiqi Wang, Xiaohua Zheng

**Affiliations:** 1 The People’s Hospital of Danyang, Affiliated Danyang Hospital of Nantong University, Danyang 212300, China; qicheng9731@gmail.com; 2 School of Pharmacy, Nantong University, Nantong 226001, China; ginyi915@163.com (J.C.); quyijie210111@163.com (Y.Q.); lx20240509@163.com (X.L.)

**Keywords:** porphyrin, covalent organic frameworks, photodynamic therapy, photothermal therapy, reactive oxygen species

## Abstract

Porphyrin’s excellent biocompatibility and modifiability make it a widely studied photoactive material. However, its large π-bond conjugated structure leads to aggregation and precipitation in physiological solutions, limiting the biomedical applications of porphyrin-based photoactive materials. It has been demonstrated through research that fabricating porphyrin molecules into nanoscale covalent organic frameworks (COFs) structures can circumvent issues such as poor dispersibility resulting from hydrophobicity, thereby significantly augmenting the photoactivity of porphyrin materials. Porphyrin-based COF materials can exert combined photodynamic and photothermal effects, circumventing the limitations of photodynamic therapy (PDT) due to hypoxia and issues in photothermal therapy (PTT) from heat shock proteins or the adverse impact of excessive heat on the protein activity of normal tissue. Furthermore, the porous structure of porphyrin COFs facilitates the circulation of oxygen molecules and reactive oxygen species and promotes sufficient contact with the lesion site for therapeutic functions. This review covers recent progress regarding porphyrin-based COFs in treating malignant tumors and venous thrombosis and for antibacterial and anti-inflammatory uses via combined PDT and PTT. By summarizing relevant design strategies, ranging from molecular design to functional application, this review provides a reference basis for the enhanced phototherapy application of porphyrin-based COFs as photoactive materials. This review aims to offer valuable insights for more effective biomedical applications of porphyrin-based COFs through the synthesis of existing experimental data, thereby paving the way for their future preclinical utilization.

## 1. Introduction

Similar to chemotherapy and radiotherapy, phototherapy has been proven to be an effective and highly promising therapeutic modality for the treatment of malignant tumors [1,2]. Due to its operational simplicity, cost-effectiveness, and non-invasiveness, phototherapy has consistently attracted the attention of numerous researchers and become an area of in-depth investigation [3]. Phototherapy can be categorized into two primary mechanisms: PDT and PTT [4]. Conventional PDT converts light energy into chemical energy, which, through energy transfer, transforms the oxygen molecules around photosensitizers into cytotoxic reactive oxygen species (ROS) [5,6,7,8]. This process oxidizes biomacromolecules within cells, inducing oxidative stress and ultimately achieving therapeutic effects [9]. PTT transforms light energy into thermal energy, causing cellular proteins or enzymes to denature at elevated temperatures, leading to apoptosis or necrosis [10,11,12]. PTT has been widely used in the treatment of a variety of diseases to date [13,14,15].

Currently, many materials that possess photodynamic or photothermal capabilities under light exposure have been identified [4]. These photoactive materials include inorganic metallic substances such as gold nanoparticles [16], tungsten [17,18], and molybdenum oxides [19], as well as organic compounds like phthalocyanine compounds [20,21], methylene blue [22,23], and porphyrin derivatives [24,25,26]. Despite their potential, inorganic metallic materials still face challenges related to biometabolism and safety. Among the organic materials, porphyrins stand out for their excellent photostability and biocompatibility [27,28]. Moreover, the molecular structure of porphyrins is easily modifiable, and they can be designed into multiligand structures, facilitating the development of multifunctional materials through coordination complexes or covalent crosslinking [29,30]. A wealth of research demonstrates that, through rational molecular synthesis and customized system design, porphyrin-based materials can simultaneously exert synergistic photodynamic and photothermal effects [30]. The combination of these two phototherapeutic mechanisms overcomes the limitations of individual treatments, making the functional design of nanoplatforms for combined PDT and PTT more straightforward [31]. It also avoids the complexity of integrating two distinct therapeutic materials into a single system and the potential time lags when applying both phototherapies concurrently [32,33]. Therefore, the design of such systems holds significant research and application value.

Covalent organic frameworks, consisting of carbon, oxygen, nitrogen, hydrogen, and phosphorus, are crystalline and reticular structures [34,35]. COFs exhibit large specific surface areas and low densities, making them extensively studied in gas separation, energy storage, electronic conduction, and catalysis [35,36]. Additionally, the organic molecular characteristics of COFs confer good biocompatibility, and their porous nature allows for the loading of appropriately sized drug molecules, enabling effective drug delivery and controlled release, thus laying the foundation for biomedical applications [37,38,39]. Notably, there has been a surge in research focusing on the preparation of porphyrin-based COFs for biomedical applications [40,41]. These studies not only highlight the safety and improved water dispersibility of hydrophobic porphyrin molecules when incorporated into COFs but also demonstrate the potential to mitigate the impact of hypoxia or heat shock proteins on the performance of porphyrin-based photosensitizers used in monotherapy. Consequently, further exploration of the structural design and phototherapeutic mechanisms of porphyrin-based COFs is of great significance.

**Figure 1:**
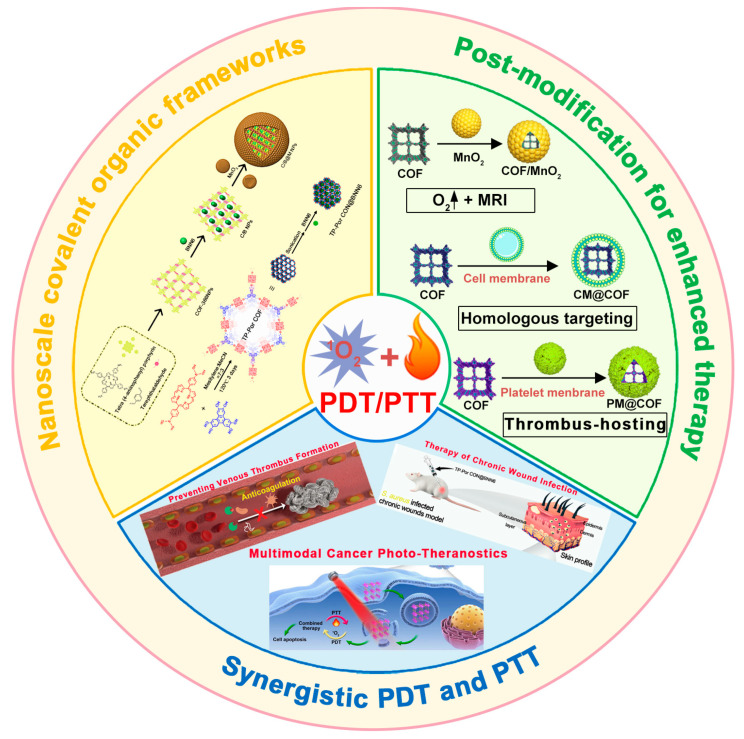
The preparation and post-modification of porphyrin-based COFs for enhanced photodynamic therapy and photothermal therapy and their application in the treatment of malignant tumors, antibacterial and anti-inflammatory conditions, and venous thrombosis. Reproduced with permission from [42,43,44,45]. Copyright (2019), Elsevier. Copyright (2021), American Chemical Society. Copyright (2022), John Wiley & Sons, Ltd. Copyright (2024), Elsevier.

This review summarizes recent advancements in the preparation and post-modification of porphyrin-based COFs for enhanced photodynamic therapy and photothermal therapy and their application in the treatment of malignant tumors, antibacterial and anti-inflammatory conditions, and venous thrombosis (Figure 1). By synthesizing and summarizing these research efforts, this review aims to provide a reference for the molecular design of nanosystems with dual phototherapeutic functionalities. It particularly emphasizes the principles behind the enhanced phototherapeutic efficacy of porphyrin-based COFs under the dual action of PDT and PTT. From the molecular architecture to functional implementation, this work offers a detailed blueprint for the biomedical application of porphyrin photosensitizers, supporting the development of more effective treatments for a variety of diseases.

## 2. Synthesis of Porphyrin-Based COFs and Their Biomedical Applications

The excellent biocompatibility and design flexibility of porphyrin molecules make them one of the most widely studied photoactive materials [28]. By reacting the free end groups of COFs with monoamino porphyrins, the loading of monoamino porphyrin molecules can be achieved for phototherapy research, without affecting the size and morphology of the COFs. This defect functionalization strategy demonstrates the feasibility and effectiveness of combining COFs with porphyrin-based photosensitizers (Table 1). Exploiting the symmetry of porphyrin molecules, photosensitizer molecules with four amino groups can be synthesized for the construction of the COF backbone. Under illumination from a single light source, these COFs can simultaneously achieve the combined functions of PDT and PTT (Table 1). The porous nature of COFs facilitates the loading of various small organic molecules. The structural characteristics of porphyrin-based COFs enable the easy realization of multimodal imaging-guided multifunctional therapeutic strategies. These features provide a scientifically sound and effective approach for the treatment of various diseases, including malignant tumors, venous thrombosis, and antibacterial/anti-inflammatory therapies (Table 1). This review summarizes the different biomedical applications of COFs prepared with various porphyrins and compiles the methods for the nanoscale processing of porphyrin-based COFs, offering valuable insights (Table 1).

## 3. Nanoscale COFs for Combined PDT and PTT

Porphyrin-based photosensitizers incorporated into nMOFs have been extensively studied in PDT research. Lin et al. demonstrated that when porphyrins are integrated into the MOF framework, the periodic and ordered arrangement of atoms within the MOF crystal structure leads to enhanced singlet oxygen generation efficiency due to the spatial separation of hydrophobic photosensitizer molecules [49,50,51]. This makes such porous materials effective carriers for the loading of hydrophobic porphyrin molecules for phototherapeutic applications against cancer cell proliferation [51]. Similarly, COFs, which are also porous and crystalline but devoid of metal elements, offer excellent biocompatibility, long-range order, and larger pore sizes, which make them another widely researched carrier [52]. However, conventional COF synthesis often requires stringent conditions, including anhydrous and anaerobic environments at high temperatures and pressures, which can hinder reproducibility and scalability [53]. Thus, developing milder COF preparation methods that can effectively load porphyrin molecules or encapsulate photoactive species within their pores is a fascinating and worthwhile area of study.

**Figure 2:**
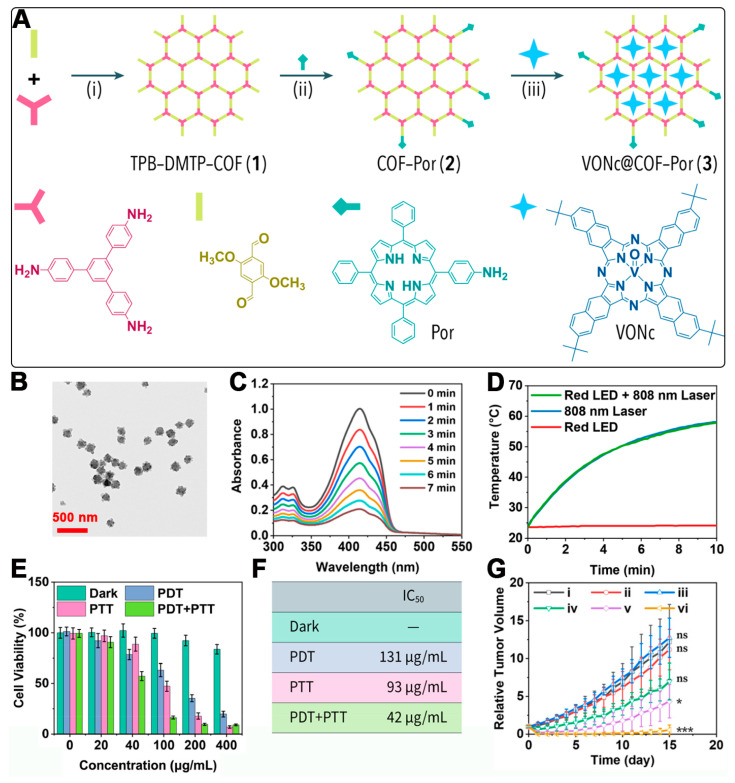
(**A**) Synthesis steps of VONc@COF-Por. (**B**) TEM images of VONc@COF-Por NPs. (**C**) ^1^O_2_ generation ability of VONc@COF-Por detected by DPBF under irradiation of a red LED. (**D**) Temperature changes in the solutions of VONc@COF-Por under red LED irradiation and/or NIR laser irradiation (808 nm). (**E**) Cell viabilities of various groups under different treatments. (**F**) IC_50_ of VONc@COF-Por under different treatments. (**G**) Relative tumor weight changes in different groups with different treatments. (i) Control, (ii) Laser, (iii) Dark, (iv) PDT, (v) PTT, (vi) PDT + PTT. (ns: no significant difference, * *p* < 0.05, *** *p* < 0.001) Reproduced with permission from [46]. Copyright (2019), American Chemical Society.

For instance, Dong et al. synthesized TPB-DMTP-COF using 1,3,5-tris(4-aminophenyl)benzene and 2,5-dimethoxyterephthaldehyde as primary precursors, with polyvinylpyrrolidone (PVP) as a dispersing agent and acetic acid as a catalyst [46]. The residual aldehyde groups on the synthesized TPB-DMTP-COF were then reacted with monoamino porphyrin to produce COF-Por, which was further used to encapsulate a phthalocyanine derivative (VONc) within its channels, resulting in VONc@COF-Por (Figure 2A). TEM images confirmed the spherical nanoparticle morphology of VONc@COF-Por, with a diameter of about 140 nm (Figure 2B). The singlet oxygen generation capability of the material was tested using 1,3-diphenylisobenzofuran (DPBF) as a singlet oxygen scavenger. Upon irradiation with red LED light (50 mW/cm^2^) for 7 min, the UV absorbance of DPBF in the presence of VONc@COF-Por decreased significantly, retaining only around 20% of its initial intensity (Figure 2C). This indicates that VONc@COF-Por effectively generated singlet oxygen under red light, leading to the degradation of DPBF. Furthermore, the photothermal conversion of VONc@COF-Por under 808 nm laser irradiation (1.5 W/cm^2^) was assessed. As shown in Figure 2D, the temperature of the VONc@COF-Por solution increased from 23.6 °C to 58.1 °C after 10 min of irradiation, whereas the control water sample showed minimal temperature change, demonstrating the material’s superior photothermal conversion performance. Given the excellent singlet oxygen generation and photothermal conversion capabilities of VONc@COF-Por, its antitumor efficacy both in vitro and in vivo was evaluated. MCF-7 cells were chosen as the target, and the cytotoxicity was assessed by an MTT assay [54,55,56]. Figure 2E illustrates that increasing concentrations of VONc@COF-Por led to the effective inhibition of cell proliferation, whether through PDT alone, PTT alone, or a combination of both. The IC50 values for PDT and PTT were found to be 131 µg/mL and 93 µg/mL, respectively. Notably, the combined PDT and PTT treatment resulted in an IC50 value of 42 µg/mL, highlighting the enhanced efficacy of the dual-modality approach, which offers the potential to achieve therapeutic outcomes at lower doses (Figure 2F). In vivo studies on MCF-7 cell proliferation in mice (Figure 2G) (administration via intratumoral injection) showed that, while individual PDT and PTT treatments initially suppressed tumor growth, the tumors eventually regrew. However, the combined PDT and PTT treatment completely inhibited tumor growth, with no increase in tumor volume observed over time, underscoring the effectiveness of the VONc@COF-Por platform in synergistic phototherapy. These results provide new insights into the design of porphyrin-based porous materials for biomedical applications and highlight the advantages of combined PDT and PTT for cancer treatment.

## 4. Nanoscale COFs for Multimodal Imaging-Guided Phototherapy

**Figure 3:**
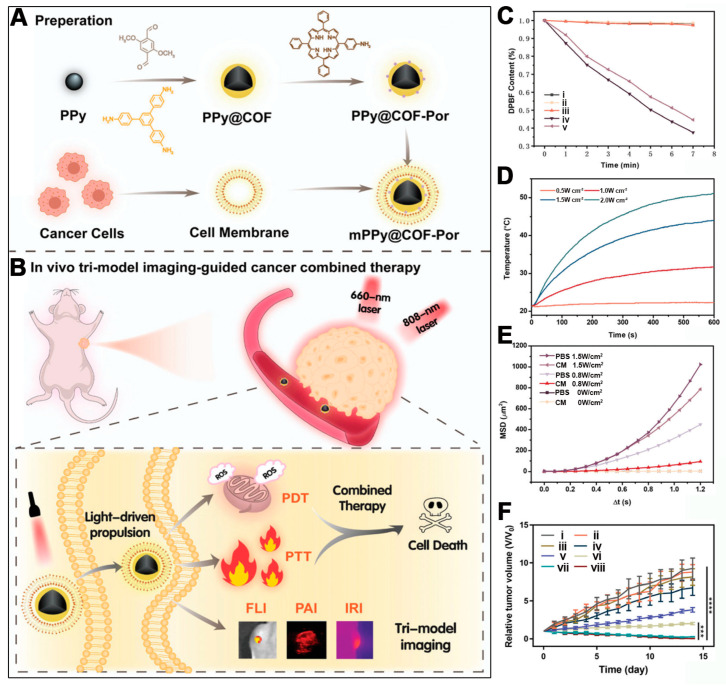
(**A**) Synthetic steps of mPPy@COF-Por nanomotor. (**B**) The mechanism of the synergistic antitumor therapy using mPPy@COF-Por nanomotors. (**C**) The ROS generation ability of the different groups after different treatments. (i) DPBF + mPPy@COF-Por + 808 nm laser, (ii) DPBF + PPy + 660 nm laser, (iii) DPBF + PPy@COF + 660 nm laser, (iv) DPBF + PPy@COF-Por + 660 nm laser, (v) DPBF + mPPy@COF-Por + 660 nm laser. (**D**) The temperature elevation induced by mPPy@COF-Por (at a concentration of 600 μg mL^−1^) under various power densities (0.5, 1, 1.5, and 2 W cm^−2^) of an 808 nm laser. (**E**) The mean square displacement (MSD) curves of mPPy@COF-Por at various power levels (0, 0.8, and 1.5 W cm^−2^) and in PBS and cell medium (CM). (**F**) Tumor volume changes in different groups with different treatments. (i) PBS, (ii) mPPy@COF-Por, (iii) PBS + 660 nm + 808 nm lasers, (iv) mPPy@COF-Por + low-power 808 nm laser, (v) mPPy@COF-Por + 660 nm laser, (vi) mPPy@COF-Por + high-power 808 nm laser, (vii) mPPy@COF-Por + 660 nm + high-power 808 nm lasers, (viii) mPPy@COF-Por + 660 nm + low-power and high-power 808 nm lasers. (*** *p* < 0.001, **** *p* < 0.0001) Reproduced with permission from [47]. Copyright (2023), John Wiley & Sons, Ltd.

Functionalizing COFs with defects and modifying the free terminal groups with monoamino porphyrins can indeed enable the loading of porphyrins for tumor photodynamic therapy. However, the inability of these COF-based photosensitive materials to specifically target cancer cells and their bioadhesion in biological media can limit the accumulation of the materials on the cancer cell surface, thus reducing the effective delivery of the photosensitizer to the tumor site. To address this, Feng et al. employed a core–shell strategy, growing COF materials, synthesized from 2,5-dimethoxyterephthaldehyde (DMTP) and 1,3,5-tris(4-aminophenyl)benzene (TPBA) monomers, onto the surface of polypyrrole (PPy) to form PPy@COF nanocomposites [47]. Subsequently, porphyrin molecules were loaded via the reactive free terminal groups of the COF, yielding the PPy@COF-Por nanoplatform (Figure 3A). To reduce the bioadhesion and enhance the targeting of cancer cells, the authors extracted HCT116 cancer cell membranes, which were then used to coat the nanoplatforms through sonication, resulting in the mPPy@COF-Por nanoplatform. The resulting nanoplatform was designed to perform multimodal imaging (thermography/photoacoustic/fluorescence)-guided 808 nm laser-activated PPy-induced PTT and red-light-activated porphyrin-induced PDT (Figure 3B). The homotypic targeting ability of the cancer cell membrane coating not only improved the targeting specificity but also reduced the adhesion in biological media, facilitating the movement of the nanoplatforms towards cancer cells. Additionally, the self-thermophoretic motion of the mPPy@COF-Por nanomotors, activated by mild 808 nm laser irradiation, promoted the directed movement of the nanoplatforms in biological media, enhancing their accumulation on the cancer cell surface. This NIR light-induced self-thermophoretic effect provides a new avenue for enhanced combined PDT and PTT.

The singlet oxygen generation capacity of the materials was assessed using DPBF as a singlet oxygen scavenger. As depicted in Figure 3C, the UV absorbance of DPBF at 413 nm decreased with prolonged 660 nm red light exposure, indicating the effective singlet oxygen generation by both PPy@COF-Por and mPPy@COF-Por. The encapsulation with cancer cell membranes did not significantly affect the singlet oxygen production. The photothermal conversion of the materials was also examined. Under 808 nm laser irradiation, mPPy@COF-Por could efficiently convert light energy into heat. The solution temperature could rise from 21.6 °C to 51.3 °C after 2 W/cm^2^ irradiation for 10 min (Figure 3D), showcasing the material’s excellent photothermal properties. The self-thermophoretic motility of the nanoplatforms in different 808 nm laser powers and media was characterized, revealing that the average squared displacement (MSD) of the mPPy@COF-Por nanomotors increased with the laser power, suggesting passive Brownian motion (Figure 3E). This 808 nm light-activated self-propulsion enhanced the accumulation of the nanoplatforms on the cancer cell surface. After confirming the PDT, PTT, and self-thermophoretic motility of the materials, the therapeutic effects of mPPy@COF-Por on HCT116 xenograft mouse models were evaluated. As shown in Figure 3F, neither PDT nor PTT alone (groups 5 and 6, respectively) could fully inhibit the continuous proliferation of cancer cells. However, the combined PDT and PTT mechanism, activated by 808 nm and 660 nm light (group 7), effectively suppressed the proliferation of HCT116 cancer cells, with only three small tumors remaining. Interestingly, in group 8, where low and high 808 nm power and 660 nm light were sequentially applied, only one small tumor remained, demonstrating the best antitumor effect. This system leverages the homotypic targeting ability of the cancer cell membrane to enhance the accumulation of the nanoplatforms on cancer cells and the NIR light-induced self-thermophoretic motion to promote the directed movement and cellular uptake of the nanoplatforms. With these enhancements, the mPPy@COF-Por nanoplatform achieved outstanding combined PDT/PTT antitumor efficacy, providing valuable preclinical data for the application of porphyrin-loaded COF-based photosensitive materials. Although the 808 nm light-activated self-thermophoretic effect may diminish for deeper tissue, the utilization of the higher levels of GSH and hydrogen peroxide in cancer cells to activate the directional movement of nanomotor drugs may be a promising future research direction.

## 5. Porphyrin COFs for Combined PDT and PTT

Defect functionalization and the reaction of free terminal groups in COFs can indeed achieve the loading of porphyrin photosensitizers. However, such designs typically result in the relatively low loading of porphyrin photosensitizers and are limited to PDT effects only. To achieve a combined PDT and PTT therapeutic effect, additional photothermal agents must be incorporated, and two different light sources are required for activation. On one hand, the use of multiple light sources complicates the treatment process and increases the cost due to the requirement for more sophisticated laser equipment. On the other hand, the non-simultaneous occurrence of PDT and PTT may prevent the synergistic mechanisms from being fully realized. Photothermal therapy can elevate the local temperature of the tumor tissue, thereby improving blood circulation and oxygen transport, which is beneficial in alleviating the hypoxia that inhibits PDT. Therefore, it is highly desirable and worth investigating to develop a single-light-source-activated photosensitive material capable of delivering both PDT and PTT.

**Figure 4:**
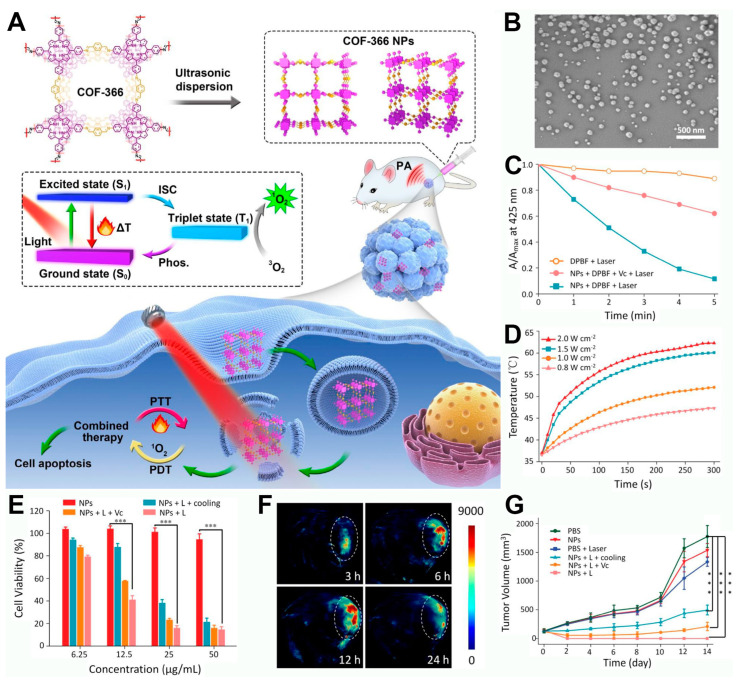
(**A**) Preparation of COF-366 NPs for PAI-guided phototherapy. (**B**) Scanning electron microscopy image of COF-366 nanoparticles. (**C**) ^1^O_2_ generation ability of COF-366 NPs after different treatments (Vc was used as the ROS scavenger). (**D**) Temperature changes in the solution of COF-366 NPs under 635 nm laser irradiation with various power densities. (**E**) Cytotoxicity of COF-366 NPs after different treatments. (**F**) Photoacoustic images of the tumor following intravenous injection at various time points. (**G**) Tumor volume changes after different treatments. (*** *p* < 0.001) Reproduced with permission from [42]. Copyright (2019), Elsevier.

To this end, researchers have envisioned the direct synthesis of porphyrin-based COFs using tetra-aminoporphyrin molecules, which would not only increase the loading efficiency of photosensitizers but also enable combined PDT and PTT treatment under a single light source. For example, Tian et al. prepared a porphyrin-based COF-366 reticular polymer via a Schiff base reaction between tetra-aminoporphyrin and terephthalaldehyde [42]. To control the morphology to a nano-sized structure, the authors employed an ultrasonic-assisted exfoliation method to obtain COF-366 nanoparticles (NPs) (Figure 4A). Under 635 nm laser irradiation, the COF-366 NPs were capable of generating singlet oxygen and converting light energy into thermal energy. Moreover, the photothermal conversion property of the prepared COF-366 NPs endowed them with the capacity for photoacoustic imaging. After cellular internalization, the COF-366 NPs could exhibit a combined PDT and PTT therapeutic effect under the guidance of photoacoustic imaging and a single light source (Figure 4A). TEM images showed that the prepared COF-366 NPs presented as nanoparticles with a relatively small but non-uniform size (Figure 4B), which was suitable for cellular uptake. The authors then used DPBF as a singlet oxygen scavenger to test the singlet oxygen generation capability of the COF-366 NPs (Figure 4C). As illustrated in Figure 4C, under continuous 635 nm laser irradiation, the UV absorbance of DPBF continuously decreased, and the extent of the decrease was mitigated upon the addition of the reductant vitamin C. These results confirmed that the COF-366 NPs can effectively generate singlet oxygen under laser irradiation. Next, the photothermal conversion efficiency of the COF-366 NPs was evaluated. As shown in Figure 4D, with the increase in the light power density from 0.8 W/cm^2^ to 2 W/cm^2^, the temperature of the COF-366 NP solution gradually increased, reaching a maximum temperature rise greater than 20 °C. These results indicate that the COF-366 NPs meet the requirements for photothermal agents. After verifying the singlet oxygen generation and photothermal properties of the material, the authors tested its cytotoxicity. As shown in Figure 4E, after cellular internalization, the COF-366 NPs under 635 nm laser irradiation could achieve a combined PDT and PTT anticancer effect. In cellular experiments, the authors inhibited the PTT effect by lowering the ambient temperature and the PDT effect by adding vitamin C. It was found that the combined PDT and PTT treatment had a significantly enhanced inhibitory effect on cell viability compared to either PDT or PTT alone. The COF-366 NPs prepared in this system have a broad absorption range, extending into the near-infrared region, suggesting that they may possess photoacoustic imaging capabilities. According to the data in Figure 4F, after intravenous injection, the photoacoustic signal intensity in the tumor region gradually increased and reached a peak at 12 h, indicating that the COF-366 NPs not only have good photoacoustic imaging characteristics but also can effectively accumulate in tumor tissue, showing their potential as tumor-targeting imaging agents. Subsequently, the authors evaluated the in vivo antitumor effect of the COF-366 NPs in a 4T1 tumor-bearing mouse model, recording the changes in the tumor volume over 14 days (Figure 4G). It was found that, compared to individual PDT or PTT, the combined PDT and PTT treatment exhibited the best antitumor effect. This result demonstrates that porphyrin-based COFs, under a single light source, can achieve complete tumor inhibition, offering a new design concept for biomedical applications of porphyrin-based COFs.

## 6. Nanoscale COFs for Combined PDT/PTT/Gas Therapy

**Figure 5:**
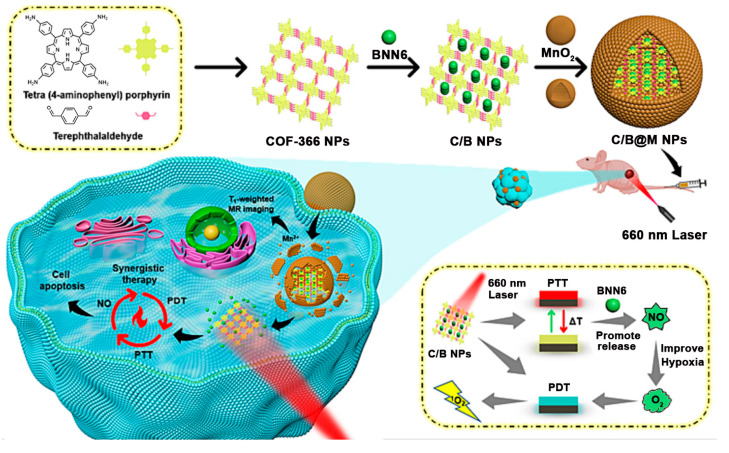
Illustrative representation of the synthesis process and integration of the C/B@M nano-delivery system for melanoma therapy. Reproduced with permission from [45]. Copyright (2024), Elsevier.

Although a single light source can activate porphyrin-based COFs for combined PDT and PTT, the hypoxic microenvironment still limits the efficacy of PDT. Therefore, employing strategies that generate oxygen can significantly mitigate the limitations imposed by hypoxia on PDT. A common approach is to use Pt NPs [57,58,59] or MnO_2_ [60,61,62,63] to catalyze the excess hydrogen peroxide within cancer cells to produce oxygen. In addition, the delivery of high concentrations of NO donor materials via carriers can also alleviate the inhibition of PDT by hypoxia [45]. High concentrations of NO not only interact with reactive oxygen species (ROS) to induce oxidative stress or nitrosative stress, ultimately leading to DNA damage and apoptosis in cancer cells, but also indirectly suppress the expression of HIF-1α and normalize the tumor vasculature, thereby reducing the impact of the hypoxic microenvironment on aerobic PDT [64,65,66]. Therefore, the integration of NO donors with PDT is a promising research direction [67,68,69]. The porous nature of COFs makes them a suitable carrier for NO donors. Based on this, Zhou et al. synthesized COF NPs using tetra-aminoporphyrin and terephthalaldehyde and then utilized the π–π stacking interactions of the COF’s conjugated structure to load the NO donor molecule *N*,*N*′-di-substituted butyl-*N*,*N*′-dinitro-1,4-phenylenediamine (BNN6), resulting in C/B NPs [45]. BNN6 has some degree of photothermal responsiveness. The authors then coated the C/B NPs with a layer of MnO_2_ to create a multifunctional combination therapy nanoplatform, C/B@M NPs (Figure 5). Under the weakly acidic conditions inside cancer cells, the MnO_2_ shell would be degraded, releasing BNN6. The photothermal effect generated by the porphyrin-based COFs under laser irradiation would then prompt BNN6 to release NO. In acidic conditions, the released Mn^2+^ ions can serve as a contrast agent for magnetic resonance imaging (MRI), which can guide the accumulation of the nanoplatform at the tumor site and determine the optimal timing for administration. Additionally, MnO_2_ can catalyze the decomposition of excess hydrogen peroxide within cancer cells to produce oxygen, thereby alleviating the hypoxic microenvironment that limits PDT. Consequently, under 660 nm laser irradiation, this system can achieve the triple-combined therapy of MRI-guided PDT, PTT, and NO-mediated gas therapy. This system takes advantage of the COF’s inherent porous structure to incorporate NO donors and the ease of surface modification to coat with MnO_2_, a catalyst that generates oxygen from hydrogen peroxide. The MnO_2_ coating serves to prevent premature singlet oxygen generation by the porphyrin-based COFs under natural light, avoiding damage to normal tissue, and to prevent the early release of NO, ensuring that NO is only released at therapeutic concentrations. Low concentrations of NO not only fail to kill cancer cells but may also promote their metastasis and proliferation. The perfect design of this system, from structure to function, provides a new concept for the enhancement of the combined PDT and PTT treatment of porphyrin-based COFs under light irradiation.

## 7. Staggered Stacking COFs for Phototherapy-Induced Immunotherapy

**Figure 6:**
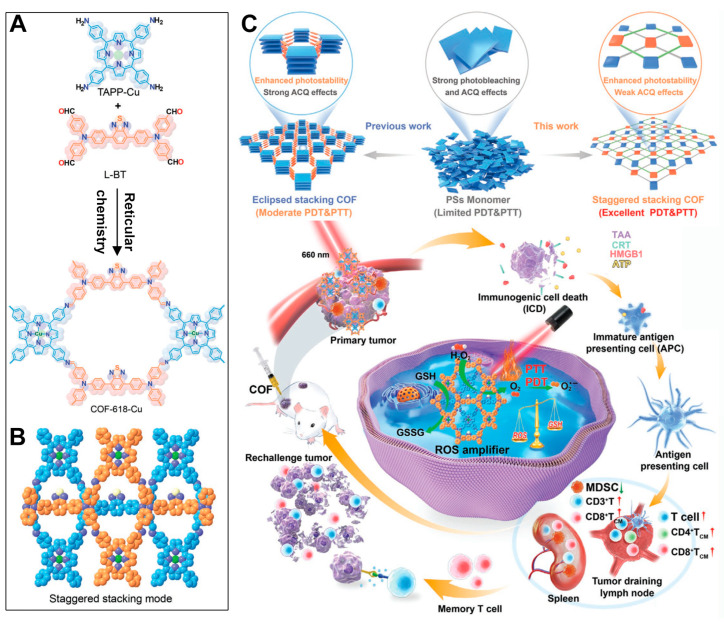
(**A**) Synthesis steps of COF-618-Cu. (**B**) Staggered stacking mode of COF-618-Cu. (**C**) Advantages of the COF-618-Cu nanoplatform compared to individual porphyrin photosensitizer molecules and porphyrin-based eclipsed stacked COFs. The combined PDT and PTT therapeutic effects of the COF-618-Cu nanoplatform, along with its induction of immunogenic cell death and synergistic mechanism with immune checkpoint blockade therapy. Reproduced with permission from [48]. Copyright (2022), John Wiley & Sons, Ltd.

PDT and PTT not only synergize with each other but also both can trigger immunogenic cell death (ICD). This therapeutic mechanism not only helps to alleviate the resistance of the tumor immune-suppressive microenvironment to immunotherapy but can also enhance the efficacy of immune checkpoint blockade (ICB) therapy, thereby achieving the effective inhibition of distant tumors and recurrent tumors. The development of a nanoplatform that integrates porphyrin-based COFs for phototherapy with its induced immunotherapeutic effects may further promote preclinical applications. For instance, Sun et al. synthesized an alternating stacked COF nanoplatform named COF-618-Cu via a solvothermal reaction using 5,10,15,20-tetrakis(4-aminophenyl)porphyrin copper(II) (TAPP-Cu) and tetra-aldehyde monomer (L-BT) (Figure 6A,B) [48]. After the validation of the crystal structure and comparison of the photo-physicochemical properties, the authors found that the prepared COF-618-Cu could achieve excellent combined PDT and PTT therapeutic effects (Figure 6C). Hydrophobic porphyrin photosensitizer molecules tend to aggregate in aqueous solutions, leading to aggregation-induced quenching (ACQ) and photobleaching, which affects their phototherapy applications (Figure 6C). While preparing porphyrins into eclipsed stacking COFs can improve the photostability of hydrophobic porphyrin molecules, strong ACQ issues still occur. Moreover, due to the high concentration of glutathione (GSH) and the hypoxic microenvironment within cancer cells, the phototherapy performance of eclipsed stacked COF materials remains challenged. Surprisingly, the staggered stacked COF-618-Cu nanoplatform not only exhibits catalase-like activity, converting excessive intracellular hydrogen peroxide into oxygen to mitigate the hypoxia limitation on PDT efficacy, but also displays glutathione peroxidase-like activity (Figure 6C). By reducing the cellular GSH concentration, it amplifies the ROS treatment effect. Under 660 nm laser irradiation, the COF-618-Cu nanoplatform demonstrates superior ROS generation capabilities, regardless of whether there are normoxic or hypoxic conditions. The combination of COF-618-Cu’s PDT efficiency and its photothermal conversion ability can induce robust ICD, remodel the tumor immune-suppressive microenvironment, and synergize with αPD-1 to inhibit the proliferation of distant tumors and the recurrence of regrown tumors. The unique and superior mechanistic process of COF-618-Cu paves a new path for the enhancement of the phototherapy applications of porphyrin-based COFs, offering a promising approach to overcoming the existing limitations and improving the therapeutic outcomes in oncology.

## 8. Nanoscale COFs for Prevention of Venous Thrombus Formation via Phototherapy

Porphyrin-based COFs exhibit excellent singlet oxygen generation under light irradiation, making them suitable for PDT. The porous structure and large specific surface area of COFs make them ideal for the loading of drug molecules, enabling multimodal imaging and combined therapeutic approaches. These multifunctional properties open up possibilities for the treatment of various diseases. Besides combating malignant tumor cells, the PDT and PTT capabilities of porphyrin-based COFs can be harnessed for the treatment of cardiovascular diseases, which pose significant health risks [44]. Among these, venous thrombosis, which is characterized by high recurrence and mortality rates, is a crucial area of medical research aimed at improving quality of life [70,71,72]. Researchers have discovered that the PTT effect can lead to the degradation of fibrin in thrombi, while PDT can prevent the subsequent blockage of blood vessels by fragmented clots [44]. Therefore, porphyrin-based COFs that combine PTT and PDT may be a promising material for venous thrombosis treatment. These materials possess good biocompatibility and excellent photostability, and their non-invasive PDT and PTT treatment modalities have been extensively studied. The non-pharmacological treatment of venous thrombosis through PDT and PTT may bring new hope to the management of this cardiovascular condition.

**Figure 7:**
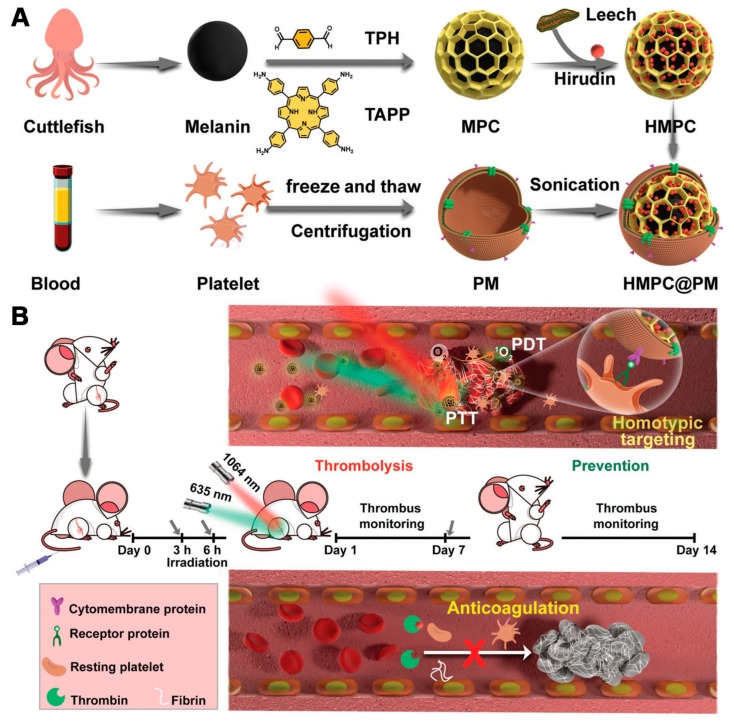
(**A**) Diagrammatic representation of the preparation method and (**B**) the mechanism of thrombolysis and prevention by HMPC@PM nanoparticles. Reproduced with permission from [44]. Copyright (2022), John Wiley & Sons, Ltd.

Against this backdrop, Li et al. developed a core–shell-structured melanin–porphyrin COF (MPC) nanoplatform by first extracting melanin from cuttlefish and then performing a Schiff base reaction between tetrakis(aminophenyl)porphyrin and terephthalaldehyde on the surface of the melanin core [44]. They subsequently loaded hirudin into the COF’s porous structure to create the HMPC nanoplatform (Figure 7A). To enhance the targeting of the thrombus, the authors isolated platelets from blood and mixed the platelet membrane with HMPC through sonication, forming the HMPC@PM nanoplatform (Figure 7A). Due to the homotypic targeting ability of the platelet membrane, the HMPC@PM nanoplatform can specifically reach the thrombus site in veins. Once at the thrombus, the HMPC@PM nanoplatform is activated by a 635 nm laser to excite the porphyrin COF for PDT, while a 1064 nm laser is used to activate the melanin core for PTT, achieving a combined thrombolysis effect (Figure 7B). The excessive heat from PTT causes the fibrin in the thrombus to fragment, and the ROS produced by PDT prevent the fragmented clots from causing secondary blockages. Notably, the hirudin loaded within the porphyrin-based COFs had a high loading rate of 97%, with slow and sustained release over 14 days (Figure 7B). Hirudin, a 65–66-amino-acid peptide, is the most potent natural specific inhibitor of thrombin. Compared to heparin, hirudin exhibits superior anticoagulant activity and significantly reduces the risk of thrombosis recurrence, without the potential side effect of thrombocytopenia. This biomimetic nanosystem presents a highly promising approach to enhancing thrombolytic therapy and reducing the bleeding risks associated with thrombotic diseases. The successful application of this system further expands the biomedical applications of porphyrin-based COFs, bringing hope to the treatment of cardiovascular diseases and representing a significant breakthrough in the medical field.

## 9. Nanoscale Por-COFs for Combined PDT/PTT/Gas Therapy in Chronic Wound Infections

Apart from malignant tumors and cardiovascular diseases, pathogen infections can also affect human health by impeding the treatment of wound-related conditions [73,74]. Microbial infections, which contribute to rising mortality rates, have become a critical focus of medical research, particularly in the development of new antimicrobial therapies [75,76]. Traditional antibiotics are prone to inducing resistance, often leading to treatment failure and the need for repeated interventions [43]. Thus, developing novel antimicrobial strategies is essential and holds significant importance in improving quality of life, especially in elderly populations. Porphyrin-based COFs, known for their good biocompatibility, have been found to exert combined PDT and PTT effects under 635 nm red light. PDT, through the generation of singlet oxygen, can selectively and effectively damage bacteria, and its low toxicity and non-invasive nature make it a promising strategy for antimicrobial applications [74]. PTT, by generating localized hyperthermia, can also induce bacterial apoptosis [75]. Therefore, porphyrin-based COFs capable of producing a combined PDT and PTT effect under light irradiation represent a highly promising class of photosensitizers for antimicrobial therapy. Moreover, the porous nature and π–π stacking interactions of COFs allow for the efficient loading of various organic small molecules, further enhancing the combined PDT and PTT therapeutic effects.

**Figure 8:**
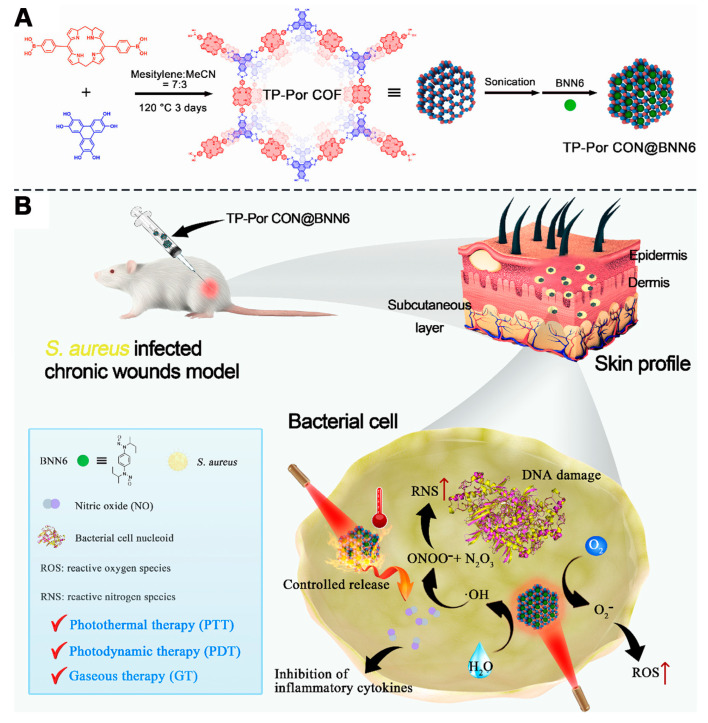
(**A**) Preparation procedures of TP-Por CON@BNN6 (TP-Por CON: porphyrin-based COF, BMM6: NO donor); (**B**) The combined application of TP-Por CON@BNN6 in the field of chronic wound infections (PDT + PTT + NO induced gas therapy). Reproduced with permission from [43]. Copyright (2021), American Chemical Society.

For example, Sun et al. synthesized boronate ester COFs (TP-Por COF) using porphyrin diboronic acid and 2,3,6,7,10,11-hexahydroxytriphenylene (HHTP) units [43]. By exploiting the exfoliable layered structure of the COFs, the authors obtained few-layer TP-Por COF nanosheets (TP-Por CON) through sonication and purification steps (Figure 8A). Subsequently, the π–π stacking interactions of the COFs were utilized to encapsulate *N*,*N*′-di-sec-butyl-*N*,*N*′-dinitro-1,4-phenylenediamine (BNN6) within the TP-Por CON, creating a multifunctional therapeutic platform, TP-Por CON@BNN6 (Figure 8A). BNN6, a NO donor, releases NO radicals upon photothermal activation. NO-mediated gas therapy enhances the combined PDT and PTT effects and plays a crucial role in wound contraction and immune responses. NO is widely used in antimicrobial treatments and can reduce inflammatory factors. The TP-Por CON@BNN6 platform, with its good biocompatibility and biodegradable boronate ester linkages, demonstrated effective antibacterial and anti-inflammatory activity under single light irradiation. The combined PDT/PTT/gas therapy provided by TP-Por CON@BNN6 significantly promoted the healing of chronic wounds infected with Staphylococcus aureus (Figure 8B). The successful application of this system once again highlights the potential of porphyrin-based COFs to treat various diseases effectively through simultaneous PDT and PTT mechanisms. These applications underscore the fact that porphyrin-based COFs are not just single photosensitizers but also highly effective carriers for drug molecules. By rationally designing the frameworks of porphyrin-based COFs to impart multifunctional capabilities, more synergistic therapeutic approaches become possible. The integration of multiple treatment modalities into a single structure combines the advantages of each while mitigating their drawbacks, opening up new possibilities for the biomedical applications of porphyrin-based COFs.

## 10. Comparative Advantages and Disadvantages of Different COF Structures for Loading of Porphyrin Molecules

In this review, the preparation of porphyrin-based COFs primarily involves two structural approaches for the loading of photosensitizer molecules. One approach is to first synthesize COFs with a nanoscale structure and then react the residual free end groups of the COFs with amine-functionalized porphyrin molecules [46,47]. This loading method does not alter the original size and morphology of the COFs. Ensuring the good water dispersibility of the COFs can, to some extent, achieve the aqueous dispersibility of the porphyrin-based COFs. Such COF carrier materials also offer the potential for large-scale production. The preparatory conditions for this optimized COF synthesis strategy are relatively mild. The porphyrin molecules loaded onto these COFs can be well separated, which is beneficial for PDT. However, this method may not achieve the high loading of photosensitizers. Furthermore, to achieve combined PDT and PTT, an additional photothermal agent and a laser source are required to activate the composite material, which may complicate the design of the composite.

The second approach involves using tetrakis(aminophenyl)porphyrin as a monomeric unit in the construction of the COF framework [42]. This preparation method can achieve the higher loading of photosensitizer molecules. The high density of porphyrin molecules allows for combined PDT and PTT effects under a single light source. Additionally, the porous structure of these COFs can be utilized to load appropriately sized organic small molecules, enabling combined treatments such as gas therapy. However, it should be noted that the synthesis conditions for these COFs are generally more stringent, requiring high temperatures, high pressures, and anhydrous and anaerobic environments, which can limit large-scale production. Moreover, the dispersibility of these COFs may be poor, necessitating intensive ultrasonication or other methods for further processing. Nonetheless, with the advancements of nanotechnology, the preparation methods for porphyrin-based COFs are becoming increasingly diverse, and their applications are expanding.

In this review, we summarize and compare the phototherapeutic activity of COFs loaded with different porphyrin photosensitizers, as well as their advantages and disadvantages in biomedical applications. However, it is important for researchers to note that the incorporation of photosensitizer molecules, including porphyrins and BODIPY molecules, into the structural composition of COFs is not a prerequisite for these materials to exhibit phototherapeutic activity. For example, Deng et al. discovered that COF-808 and COF-909, formed from certain non-photosensitive tetra-aldehyde precursors linked with p-phenylenediamine molecules, can also exert photodynamic activity under light irradiation [77]. This is because the conjugated structure of COFs can narrow the gap between the frontier orbitals of non-photosensitive molecules, thereby facilitating efficient electron–hole separation. The charge transfer process promotes the formation of superoxide and hydroxyl radicals from oxygen molecules. This provides new insights into the phototherapeutic applications of COFs.

## 11. Conclusions

This review summarizes recent advancements in the application of porphyrin-based COFs for the combined photodynamic and photothermal therapy of malignant tumors and venous thrombosis and for antibacterial treatment and wound healing. As a class of purely organic crosslinked polymers, COFs offer excellent biocompatibility [78,79,80]. The periodic and ordered arrangement of porphyrin molecules within the COF framework provides an effective carrier strategy for the targeted delivery of hydrophobic porphyrin molecules to lesion sites [52]. This review encapsulates the design concepts and the photophysical and photochemical advantages of systems that utilize dual light sources for combined PDT and PTT. Additionally, it discusses the synergistic mechanisms when PTT and PDT are simultaneously activated by a single light source, offering crucial insights for the biomedical application of porphyrin-based COF nanoplatforms.

Despite the extensive research on porphyrin-based COFs for disease treatment, several challenges remain, limiting their broader biomedical application and clinical translation. (1) The synthesis of most COFs still requires harsh conditions, including high-temperature, high-pressure, and anhydrous and anaerobic environments, which pose limitations for large-scale biomedical applications. (2) Porphyrin photosensitizers typically exhibit weak molar extinction coefficients at their maximum absorption wavelengths (640–660 nm), which may limit the energy conversion efficiency of the corresponding photoactive materials. Developing porphyrin-based COFs with enhanced molar extinction coefficients is a promising direction for future research [30]. (3) Most porphyrin-based COFs are synthesized using amide linkages, which may require a long time to degrade within cells [35,81]. Therefore, the design and synthesis of porphyrin-based COFs should consider their biodegradability. For instance, boronate ester-linked porphyrin-based COFs could serve as a viable alternative [43,82]. (4) Although porphyrin-based COFs exhibit excellent photochemical properties, the poor targeting ability of these nanoplatforms remains a critical issue. Integrating these materials with biomimetic structures, such as cell membranes, could enhance their targeting capabilities [47,83]. However, the safety of such structures must be carefully evaluated before use. (5) The combination of immunotherapy and tumor therapy can bring new hope for tumor treatment [84,85]. Both photodynamic therapy and photothermal therapy can induce immune effects through different mechanisms. Therefore, further research on the immune effects caused by porphyrin-based COFs during phototherapy may further enhance the elimination of diseased tissue. (6) Despite the improvement in the aqueous dispersion stability of hydrophobic porphyrin molecules by incorporating them into COFs materials, it is important to note that the stacking interactions between layers within the COF structure can still lead to the significant aggregation-induced quenching of some porphyrin molecules. This phenomenon results in the phototoxicity of porphyrin-based COFs being lower than that of individual porphyrin molecules. Developing more sophisticated and rationally designed porphyrin-based COFs could further advance the preclinical applications of porphyrin photosensitizers. Porphyrin-based COFs are relatively new biomedical nanomaterials, and more in-depth medical research is needed, supported by additional data. This review provides a valuable reference for the biomedical application of these materials, highlighting the importance of addressing the current challenges to unlock their full potential.

## Figures and Tables

**Table 1:** Porphyrin COFs for synergistic PDT and PTT applications.

Material	Structure		Function/Therapeutic Advantages	Ref
	Porphyrin	Nanoscale COF		
VONc for PTT Porphyrin for PDT VON@COF-Por	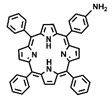	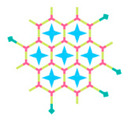	PDT + PTT Red LED (PDT), 808 nm (PTT), MCF-7 tumor cell	[46]
PPy for PTT mPPy@COF-Por	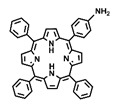	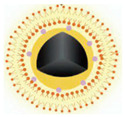	PDT + PTT, 660 nm (PDT), 808 nm (PTT), homotypic targeting, HCT116 cancer cells	[47]
Ultrasonic dispersion COF-366	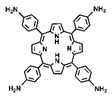	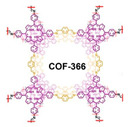	PDT + PTT 635 nm, PA imaging, 4T1 cancer cells	[42]
BNN6 (NO) MnO_2_ C/B@M NPs	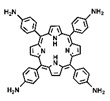	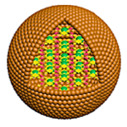	PDT + PTT + gas therapy (NO), 660 nm, MR imaging, O_2_ generation, B16F10 cancer cells	[45]
COF-618-Cu	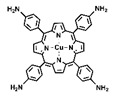	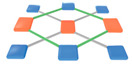	PDT + PTT, GSH depletion, O_2_ generation, immunogenic cell death, CT26 tumor	[48]
Melanin for PTT Platelet membrane HMPC@PM	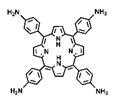	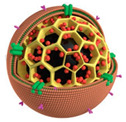	PDT + PTT + thrombolysis (Hirudin), 635 nm (PDT), 1064 nm (PTT), thrombus-hosting properties, preventing venous thrombus formation	[44]
BNN6 (NO) TP-Por CON@BNN6	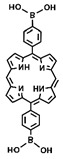	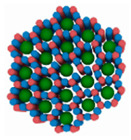	PDT + PTT + gas therapy (NO), 635 nm, destroyed the bacterial cells, therapy for chronic wound infections	[43]

Abbreviations: VONc (naphthalocyanine); PPy (polypyrrole); BNN6 (NO donor, *N*,*N*′-disubstituted butyl-*N*,*N*′-dinitro-1,4-phenylenediamine); HMPC (hirudin-loaded melanin–porphyrin COF).
